# Glycogen synthase kinase 3 beta: can it be a target for oral cancer

**DOI:** 10.1186/1476-4598-9-144

**Published:** 2010-06-11

**Authors:** Rajakishore Mishra

**Affiliations:** 1 Dept. of Molecular Pharmacology and Therapeutics, Loyola University Medical Center, 2160 South First Avenue, Bldg 102, Maywood, IL-60153, USA

## Abstract

Despite progress in treatment approaches for oral cancer, there has been only modest improvement in patient outcomes in the past three decades. The frequent treatment failure is due to the failure to control tumor recurrence and metastasis. These failures suggest that new targets should be identified to reverse oral epithelial dysplastic lesions. Recent developments suggest an active role of glycogen synthase kinase 3 beta (GSK3 β) in various human cancers either as a tumor suppressor or as a tumor promoter. GSK3β is a Ser/Thr protein kinase, and there is emerging evidence that it is a tumor suppressor in oral cancer. The evidence suggests a link between key players in oral cancer that control transcription, accelerated cell cycle progression, activation of invasion/metastasis and anti-apoptosis, and regulation of these factors by GSK3β. Moreover, the major upstream kinases of GSK3β and their oncogenic activation by several etiological agents of oral cancer support this hypothesis. In spite of all this evidence, a detailed analysis of the role of GSK3β in oral cancer and of its therapeutic potential has yet to be conducted by the scientific community. The focus of this review is to discuss the multitude of roles of GSK3β, its possible role in controlling different oncogenic events and how it can be targeted in oral cancer.

## Introduction

Oral cancer is the sixth most common cancer in the world, and its incidence varies in different ecogeographic regions [1,2]. Its occurrence is associated with exposure to smoking and alcohol consumption in the Western population. The majority of cases occur in Asia, where it is mainly associated with betel quid chewing [3]. Poor oral hygiene and human papillomavirus (HPV) infection of oral epithelial cells are other etiological factors [4]. In addition to genetic differences, other etiological factors promote the occurrence of this disease to different extents in different populations. Although there are several differences in disease occurrence and etiology between populations, there is one aspect of these tumors that is highly similar worldwide. Oral tumors are mainly asymptomatic initially, are aggressive, and frequently invade and migrate to distant organs, making them difficult to treat. This suggests that, although different predisposing factors activate various molecular pathways [5], eventually all of them may follow a common path thereafter to result in oral cancer.

Advances in recent decades in the surgical, radiotherapeutic and chemotherapeutic treatment of oral cancer have only modestly improved patient survival. Various approaches have been used for the clinical treatment of oral cancer patients in the last three decades, from non-targeted chemotherapy to highly targeted pharmacological inhibitors and specific monoclonal antibodies [3,6]. Although targeted therapies yield better outcomes than non-targeted therapies, frequent treatment failure suggests the need for new treatments or targets for this disease. In oral cancer, active transcription of various genes leads to rapid cell division, faster invasion and reduction of cell death. Although it has been largely overlooked, there is a potential link between key players in oral cancer, including transcription factors, cell cycle regulators, invasion/metastasis-promoting factors, and cell survival regulators, and their regulation under the control of glycogen synthase kinase 3β (GSK3β).

GSK3β plays a major role in epithelial cell homeostasis [7]. Its activity is regulated by site-specific phosphorylation of Tyr216/Ser9 residues [8]. The regulated phosphorylation of Ser9GSK3β is the main cause of various pathological conditions, and it is upregulated in epithelial cancers. Many upstream kinases protein kinase A (PKA) [9], Akt/PKB [10], PKC [11], p90 ribosomal S6 kinase/MAPK-activating protein (p90RSK/MAPKAP) [12] and p70 ribosomal S6 kinase (p70S6K) [13] are known to phosphorylate Ser9 of GSK3β, depending on the cellular context and various upstream regulators. The oncogenic activation of these upstream signaling molecules is frequently reported in oral squamous cell carcinoma (OSCC) [14-16]. Many of these oncogenic pathways are activated by common etiological factors of this cancer. Overall, this evidence suggests the possible active involvement of GSK3β-mediated signaling in this neoplastic disease. This review attempts to correlate the established pathways of oral cancer with GSK3β signaling and discusses the potential of this kinase as a therapeutic target.

### The GSK3 family and its regulation

GSK3 was discovered nearly three decades ago in rabbit skeletal muscle as a protein kinase that phosphorylates and inactivates glycogen synthase, the final enzyme of glycogen biosynthesis [17,18]. GSK3 is a multifunctional Ser/Thr kinase with diverse roles in various human diseases, including diabetes, inflammation, neurological disorders and various neoplastic diseases [19,20]. To date, two members of the mammalian GSK3 family (α and β) are known [18]. They are ubiquitously expressed and highly conserved and are members of the CMGC family of protein kinases [21]. Many of the substrates of GSK3 need a "priming phosphate" (which is a Ser/Thr residue) located four amino acids (aa) C-terminally from the site of phosphorylation [8]. GSK3 is constitutively active in resting cells and undergoes a rapid and transient inhibition in response to a number of external signals. Physiological regulation of GSK3 activity by various upstream kinases [9-13] in different physiological and pathological condition is established [8].

### GSK3β and its role in tumorigenesis

GSK3β drives oncogenic progression either by its inhibition or its activation, depending on the cell type. In recent years, its role in cancer has become firmly established. The differences in the roles of GSK3β depending on the type of cancer are quite interesting. Whereas it has a growth-promoting role in some cancers, it suppresses growth in others. Based on the literature, it is clear that GSK3β can act either as a tumor promoter or as a tumor suppressor, as shown in Table 1.

**Table 1:** Paradoxical role of GSK3β in various human cancers

Cancer Types	Explanation for Tumour Suppressor Role of GSK3β
Skin cancer (Cutaneous SCC)	Inactivation of GSK3β (higher pSer9GSK3β expression) [72] Inactivation of GSK3β (lower pTyr216GSK3β expression) [60,168] Pharmacological inhibition of GSK3β in normal epithelial causes epithelial mesenchymal transition (EMT) and invasion [39]

Oral cancer (OSCC)	Inactivation of GSK3β (higher pSer9GSK3β expression) [88] The basal inactivated GSK3β (pSer9GSK3β) level in OSCC cell line is high [61-63] Activation of GSK3β, can reverse EMT [64]

Larynx cancer	Inactivation of GSK3β (higher pSer9GSK3β expression) [88]

Esophageal cancer	Inactivation of GSK3β (higher pSer9GSK3β expression) [88]

Breast cancer	Overexpression of inactive GSK3β promotes [169], and active GSK3β suppress mammary tumours [168] Active GSK3 increases chemosensitivity, cell cycle arrest and reduces mammary tumorigenecity [170-172] Pharmacological inhibition of GSK3 in breast epithelial causes EMT and invasion [39]

Salivary gland cancer	Inactivation of GSK3β (pSer9GSK3β) observed in this tumor [88]

Nasopharyngeal cancer (SCC)	Inactivation of GSK3β observed and positively correlated with its upstream inactivating kinase Akt [173]

Lung cancer (SCC)	Inactivation of GSK3β reported [40]

Adenocarcinoma of Lung	Higher level of inactivated of GSK3β (pSer9GSK3β) observed [174]

Melanoma cancer	Inactivation of GSK3β reported [60]

Skin cancer (Basal cell carcinoma)	Inactivation of GSK3β reported [60]

**Cancer Types**	**Explanation for Tumour Promoter Role of GSK3β**

Pancreatic cancer	Pharmacological inhibition of GSK3 attenuates survival, proliferation and induce apoptosis [162,163,175] Active GSK3β promotes growth [176] Absence of inactive GSK3β (lower pSer9GSK3β expression) in tumors [88] High level expression and nuclear accumulation association with kinase activity and tumor dedifferentiation [161,177,178]

Colorectal cancer	Pharmacological inhibition activates cell cycle arrest and induce apoptosis [158,159,175] Absence of inactive GSK3β (lower pSer9GSK3β) in majority of tumors [88] Increased expression/active GSK3β in these tumors [88,159]

Myeloma cancer	GSK3β promotes growth and use of pharmacological inhibitor promotes apoptosis [83]

Hepatic cancer	Absence of inactive form of GSK3β (pSer9GSK3β) in these tumors [88] Increase and active GSK3β expression [175]

Leukemia cancer	GSK3 activation enhances proliferation and survival [160,179-181] Missplicing at the kinase domain causing active GSK3β [179]

Stomach cancer	Absence of inactive GSK3β (pSer9GSK3β) in these tumours [88] Active GSK3β observed frequently and its pharmacological inhibition attenuates survival, proliferation and induce apoptosis [175]

Ovarian cancer	GSK3β expression increases and it promotes cell division [156]

Prostate cancer	GSK3 activity favors replication of DNA and S-phase progression [157]

Thyroid cancer	Inhibition of GSK3 activity leads to growth suppression [182]

Gastro-Intestinal cancer	Higher and active GSK3β expression observed [166] Absence of inactive GSK3β (pSer9GSK3β) in these tumors [88]

Renal cell carcinoma	Activation of GSK3β observed in this tumor [175] Nuclear accumulation of GSK3β and its pharmacological inhibition suppress growth [178]

Glioma cancer	Pharmacological inhibition of GSK3 induces cell death [183]

### GSK3β and its control over transcription

Alteration of the transcriptional machinery is common in neoplastic diseases, including oral cancer [22,23]. Oncogenic transcription factors (OTFs) alter the transcriptional machinery to regulate mRNA synthesis. GSK3β regulates the stability of various oncogenic TFs like the activator protein 1 (AP-1) [24], nuclear factor kappa B (NFκB) [25], c-Myc [26], β-catenin [27], Snail [28], Forkhead (FH) [29], CAAT-enhancer binding protein (C/EBPs) [30], and cAMP response element-binding (CREB) [31] by phosphorylation [8]. Most of these TFs are physiological targets of GSK3β that undergo proteasomal degradation upon phosphorylation [8,24-28]. AP-1 transcriptional activity is high in oral cancer tissue samples [2]. Active GSK3β directly phosphorylates c-Jun at Thr239 which promotes its degradation [24]. It is also known that in normal oral mucosa c-Jun is localized in the cytoplasm while it enters to the nucleus at the onset of oral carcinogenesis [32]. Both Fos and Jun are phosphorylated and activated by mitogen activated protein kinase (MAPK) and c-Jun n-terminal kinase (JNK) kinase system [33,34] may be due to inactive GSK3β. Moreover the expressions of p65 (one of the NFκB family member) have been observed in oral cancer tissue samples [35,36] and metastatic OSCC [36]. GSK3β phosphorylates p65 at Ser468 and negatively regulate its activity by promoting its degradation [25]. p65 might escape from its turnover because of inactivated GSK3β in OSCC. Recent report suggests active GSK3β physically interact with IκBα in normal epithelial cells [37]. Moreover study in different system suggests that active GSK3β blocks NFκB dependent transcription, by preventing IκBα degradation [38]. In normal epithelial cells NFκB activity is known to be inhibited by GSK3 [39]. From all these evidences, it seems like NFκB activation in OSCC may be modulated, because of inactive GSK3β like that in other epithelial cancers [40]. On the other hand, degradation of c-Myc and β-catenin is initiated by phosphorylation of GSK3β [26]. The overexpression of c-Myc and β-catenin protein in OSCC is established [41-46]. The gene mutation on hot spots i.e. Thr58 of c-Myc and Ser33, Ser37, Thr41 and Ser45 of β-catenin abolishes phosphorylation by GSK3β results in preventing ubiquitination and proteasome mediated degradation of c-Myc [47-50]/β-catenin [46,51-53] has been reported in various cancers but not so far in OSCC. In OSCC, c-Myc/β-catenin protein might get stability not because of missense mutation at these hot spot codons but because of inactivation of its phosphorylating kinase i.e. GSK3β it self. The activated Snail has been reported in OSCC [54]. GSK3β is well known regulator of Snail which phosphorylates and that leads to Snail nuclear export and deregulation [28,39,55,56]. Moreover, p53 is highly involved in OSCC [57]. Though it is inactivated by mutation in nearly half of oral cancer population [57] the cause of its inactivation is still doubtful in the other half. p53 activity is regulated by active GSK3β, due to either physical association or phosphorylation and post-translational modification [58,59]. It is possible that in OSCC cases without p53 mutations [57], p53 can be inactivated due to inactive GSK3β. These OTFs those are important in OSCC and are directly regulated possibly by GSK3β. Alteration of these TFs plays a vital role in various diseases, including OSCC.

### GSK3β is a key player in OSCC

GSK3β can promote or suppress growth in different types of cancer (Table 1). The inactivation of GSK3β has been reported in most cancers of epithelial origin, such as skin, breast, and in cancers of the oral cavity, salivary glands, larynx, and esophagus [60]. The basal level of inactivated GSK3β (pSer9GSK3β) in OSCC cell lines is very high [61-63] but can be decreased by inhibiting the GSK3β upstream inactivating pathway [61,62]. A recent report suggests that activating GSK3β can reverse the epithelial-mesenchymal process in oral cancer [64]. GSK3β-mediated signaling could explain numerous molecular disorders specific to oral cancer.

#### A) Cell cycle regulation

**Figure 1:**
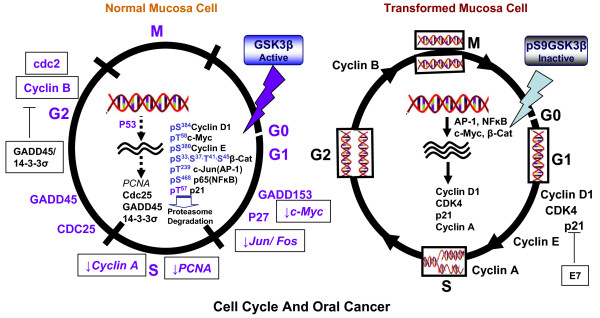
**Progressive inactivation of GSK3β may promote accelerated cell cycle and oral cancer.** As discussed in the text, most of the cell cycle regulators and their gain of function may be because of inactivation of GSK3β in oral cancer. GSK3β regulates the activity or turnover of several master cell cycle regulators like p53. Activation of p21, 14-3-3σ and GADD45 protein by p53 induces cell cycle arrest to prevent the propagation of mutations, which accumulate in cells under genotoxic stress. p53 induces the expression of the cytoplasmic scaffold protein 14-3-3σ, which prevents the nuclear import of cyclin B1 and cdc2 by sequestration in the cytoplasm. On the other hand, GADD45 destabilizes CDC2/cyclinB complexes. GSK3β-regulated c-Myc is a master regulator of the cell cycle and is essential for G0/G1-to-S progression. Myc suppresses the expression of cell cycle checkpoint genes (GADD45, GADD153) and inhibits the function of CDK inhibitors. Myc also activates cyclins D1, E1, and A2, CDK4, CDC25A, and E2F-1 and -2. Cyclin D1 is a crucial cell cycle regulator mainly regulated by the activity of TFs (NFκB, β-catenin-TCF/LEF, AP-1) and is indirectly controlled by GSK3β. Moreover, inactivation of GSK3β leads to the stabilization of cyclin D1. Oncogenic gains of function of these molecules stemming from inactive GSK3β have been established in various neoplastic diseases and might orchestrate cell cycle dysregulation in OSCC.

Cell division is a precisely regulated process that occurs obligatorily in all organisms. The ability of cells to divide is mainly attributed to the presence of three classes of molecules: CDKs (Cyclin Dependent Kinases, a family of Ser/Thr kinases), their binding partners cyclins and CDK inhibitors (CDKI) [65]. The transcriptional and post-translational regulation of cyclin D1 [66,67] and of cyclin E [68,69] in OSCC are well documented. Cyclin D1/E transcriptional upregulation is achieved by regulating TFs (e.g., AP-1, NFκB, β-catenin), and protein stability/nuclear accumulation are also increased [70,71] in OSCC [66,68,69]. Inactive GSK3β prevents the phosphorylation of Thr286 cyclin D1 and Ser380 cyclin E, which blocks their nuclear export and degradation [70-72]. An inverse correlation between cyclin D1 and GSK3β expression has been reported in oral cancer [73]. Cyclin A and cyclin B are also overexpressed in OSCC [69,74,75]. These cyclins are primarily regulated by c-Myc and p53 and thus qualify as GSK3β targets. Because these are S phase- and G2-M phase-specific cyclins, their expression is affected by the G1 phase-specific cell cycle events of cyclin D1/CDK4 and cyclin E/CDK2 activation [57,76]. Overexpression of CDK4 mRNA has been reported in different malignancies, including oral and epithelial cancer [77,78]. c-Myc controls the expression of CDK4 by binding to E-box elements present in its promoter that are not only overexpressed in OSCC [42] but also are regulated by GSK3β [26]. p21 (WAF1/CIP1) competes with cyclins for binding to CDKs, and its expression is usually decreased in various cancers. However, in OSCC, the overexpression of p21 (WAF1/CIP1) is quite evident [79], and its overexpression significantly correlates with tumor size, lymph node involvement and clinical stage [79,80]. Active GSK3β directly regulates p21 expression by phosphorylation at Thr57 [81], leading to proteasome-mediated degradation. Another explanation could be that the TFs C/EBPα and -β (which may also be stabilized because of inactive GSK3β in OSCC) interact with p21 and protect it from degradation. The possible explanations for why p21 does not halt OSCC progression are numerous. One possible explanation is that p21 is inactivated by binding to the E7 protein of human papillomavirus 16 (HPV16), which is highly prevalent in OSCC. This association of p21 and E7 blocks the ability of p21 to inhibit cyclin/CDK activity as well as PCNA-dependent DNA synthesis. In contrast, another CDKI, p27, is reportedly down-regulated in OSCC [82] in a process that might be mediated by forkhead (FH) TF [29,83]. In breast cancer (where active GSK3β acts like a tumor suppressor as in OSCC; Table 1) knock down of PI3K promotes degradation of FH and p27 possibly via GSK3β activation [84]. GADD45 and GADD153 are checkpoint inhibitors and tumor suppressors that have roles in multiple tumor types, including OSCC [85,86]. GADD45 is also controlled by p53, and upon DNA damage, it is activated to arrest the cell cycle. Both GADD45 and GADD153 are downstream targets of c-Myc [87] and thus qualify as possible GSK3β targets in OSCC. Cell division cycle 25A (CDC25A) is also controlled by c-Myc [69,76]. Direct evidence suggests a positive correlation between pSer9GSK3β and CDC25A expression in tumors of the oral cavity, salivary glands and larynx (Ref. [88] and Fig 1).

#### B) Nodal invasion by epithelial-mesenchymal transition

OSCC is a cancer of epithelial cells that invades surrounding tissues and frequently migrates to distant organs (metastasizes) [89]. The extra cellular matrix (ECM) interaction is important for the survival of normal epithelial cells but this interaction is gradually lost in squamous cell carcinoma [90]. The major ECM molecules implicated in OSCC development include collagen, fibronectin [91], tenascin [92] and laminin [54,91,93]. Many ECM molecules are indirect targets of GSK3β via Snail- or AP-1 [28,94]. The degradation of basement membrane (collagen) by MMPs and its regulation by inactive GSK3β have been reported [95,96]. Focal adhesion kinase (FAK) is overexpressed in preinvasive and invasive OSCC [97]. Upregulation of FAK leads to migration, and its regulation by active NFκB is known in tongue squamous cell carcinoma cells (SCC25) [98,99] possibly via inactive GSK3β. Another group of molecules, the integrins, are transmembrane, heterodimeric, cell-surface proteins (consisting of one α and one β subunit) that primarily function as cell adhesion molecules but also participate in signal transduction leading to cell migration, growth and oncogenesis. Human integrins are upregulated in OSCC [100,101], and they are primarily controlled by those transcription factors regulated by GSK3β [102-104]. Recent evidence suggests a role for Snail in controlling multiple α/β-integrins and EMT in OSCC [54,94,105].

MMPs are a group of extracellular matrix/basement-degrading proteases. High levels of MMP-2, -3, and -9 have been associated with poor prognosis for patients with oral cancer, including the development of lymph node metastasis and poor survival [100,106,107]. The transcriptional activation of MMP-1,-3, and -9 is common in OSCC [108,109], and they are all targets of AP-1, NFκB, C/EBPs or Snail, highlighting the importance of GSK3β-mediated signaling in the oral cancer invasion program [110-112].

**Figure 2:**
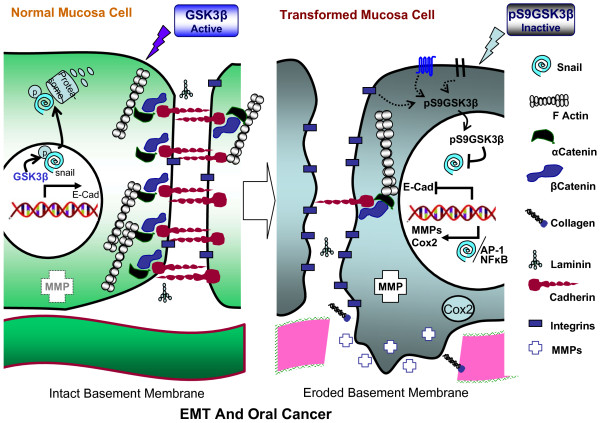
**Progressive inactivation of GSK3β may promote enhanced EMT and oral cancer.** GSK3β regulates several molecules that participate in epithelial-mesenchymal transformation, invasion and metastasis in cancer. Normal epithelial cells are connected to each other by E-cadherin, which binds to α- and β-catenin, which in turn connect E-cadherin to the actin cytoskeleton. Levels of E-cadherin are decreased in EMT. E-cadherin expression is suppressed by Snail. MMPs degrade the BM and facilitate the migration of cancer cells. Several MMPs upregulated and activated in OSCC are controlled by TFs such as Snail, AP-1, and NFκB. All of these events are directly or indirectly linked to the inactivation status of GSK3β.

Cadherins interact with the actin cytoskeleton to maintain tissue architecture. In some cancers, including OSCC, loss of E-cadherin favors invasion. An inverse correlation between E-cadherin and Snail expression has been reported in OSCC and epithelial cancers [113-115], which supports the regulation of E-cadherin by the inactivation of GSK3β and Snail [28,64]. Snail represses E-cadherin gene expression in epithelial tumours [116]. GSK3β is well known regulator of Snail which phosphorylates and that leads to Snail nuclear export and deregulation [28,39,55,56]. Recent findings suggest that the forced activation of GSK3β and the resultant phosphorylation and cytoplasmic translocation of Snail lead to E-cadherin up-regulation, which can potentially reverse EMT in OSCC [64]. Yang et al. have shown that EMT phenotypes can be decreased in head and neck SCC (HNSCC) by the use of siRNA-mediated repression of Snail or by the use of inhibitors of PI3K, which is a GSK3β-inactivating upstream kinase [90]. On the other hand, elevated Cox-2 levels have been reported in various human malignancies, including OSCC [117-119]. Inhibition of Cox-2 decreases integrin and MMP levels as well as the invasiveness of OSCC [118,119]. Cox-2 gene transcription is controlled by wild-type p53 protein [120] and by NFκB in betel quid-associated oral cancer [121], indirectly supporting the importance of inactive GSK3β (Ref [122] and Fig 2).

#### C) Anti-Apoptosis

The inhibition of apoptosis is a major cause of neoplastic disorders and an integral part of oral cancer pathogenesis. Abundant evidence suggests a possible role for active GSK3β in cell survival and apoptosis [123,124]. Apoptosis is controlled by either the intrinsic (mitochondrial) or extrinsic pathway (activation of procaspase-8) [123,125-128].

Higher levels of Bcl-2 and lower levels of Bax are frequently reported in oral cancer [127]. A recent report suggests that, in an OSCC cell line, Bcl-2 expression is affected even by slight changes in the status of pSer9GSK3β [63]. Active GSK3β blocks CREB-dependent expression of the anti-apoptotic protein Bcl-2 [128]. Additionally, active GSK3β regulates p53 activity, which increases Bax protein levels to initiate apoptosis [125]. Modulation of GSK3β can markedly increase p53-dependent activation of Bax, leading to cytochrome *c *release, loss of mitochondrial membrane potential and caspase-9 processing [125]. Moreover, the physiological effect of p53 is governed by inactivation of GSK3β (pSer9 GSK3β) [125] (and not by pTyr216GSK3β). Inhibition of Akt (a well-known kinase upstream of GSK3β) can only induce tumor necrosis factor-related apoptosis-inducing ligand (TRAIL) -mediated apoptosis by regulating the levels of Bcl-2 and Bax in OSCC [125]. All of this evidence suggests that the survival advantage of OSCC cells over the normal oral epithelium might be due to progressive inactivation of GSK3β, which could be responsible for an increased Bcl-2/Bax protein ratio [63,125-127].

**Figure 3:**
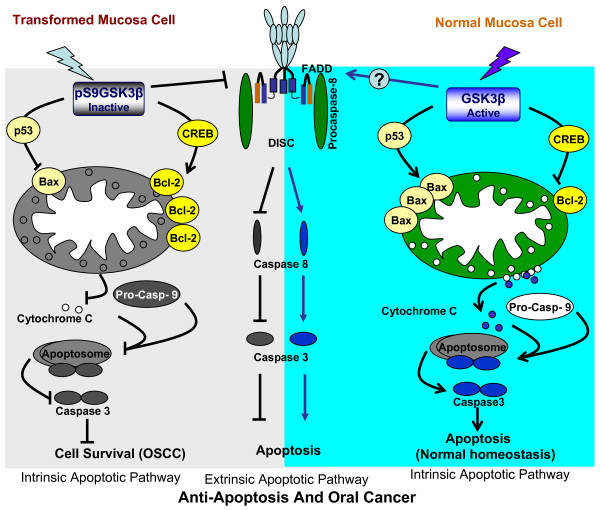
**Progressive inactivation of GSK3β may promote increased anti-apoptosis and oral cancer. **GSK3β-mediated signaling controls apoptosis in OSCC. In the intrinsic apoptotic pathway, inactive GSK3β fails to promote apoptosis by the disruption of mitochondrial membrane potential resulting from disruption of the Bcl-2/Bax ratio. Overexpression of Bcl-2 and suppression of Bax occur frequently in OSCC. This may be due to either inactive p53 (in the subgroup of cases in which p53 is not mutated or silenced) or active CREB; both are controlled by GSK3β. In the extrinsic pathway, active GSK3β promotes apoptosis by inducing procaspase-8 activation. Moreover, the inactivated GSK3β might send survival signals via the extrinsic pathway by blocking procaspase-8 activation in OSCC. By doing this, GSK3β might maintain the balance between proliferation and death and contribute to tissue homeostasis in normal oral epithelium; these might be perturbed in OSCC.

On the other hand, oral cancer cells are resistant to cell death mediated by TRAIL [126], which can be achieved only by inactivation of the GSK3-inactivating PI3K/Akt pathway [127]. Additionally, inhibition of caspase-8 reduces PI3K inhibitor-mediated apoptosis in OSCC [127]. In the extrinsic apoptotic pathway, active GSK3β promotes the activation of the initiator caspase-8 [122]. Therefore, active GSK3β targets both intrinsic and extrinsic pathways to maintain control over growth and proliferation in normal epithelium by promoting apoptosis [Fig. 3]. This control might be disrupted in OSCC.

### Oral cancer therapy and role of GSK3β signaling

**Figure 4:**
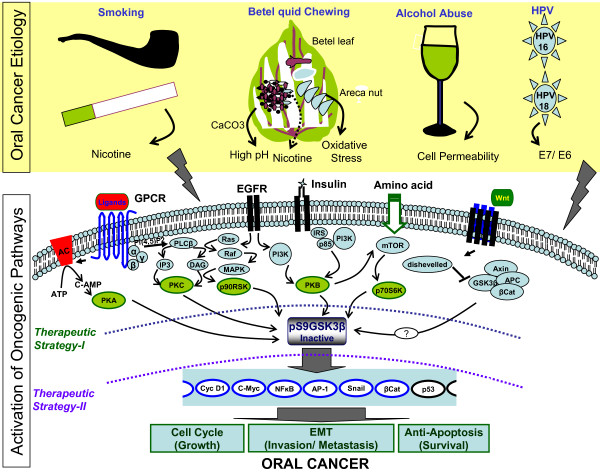
**Targeting GSK3β pathway may be highly beneficial for curing oral cancer**. Inhibition of GSK3β activity by the activation of several oncogenic pathways in cancer as discussed in the text. Activation of these pathways by several oral cancer etiological factors is interesting and fuel for inactivating GSK3β by targeting its inactivating pathways to promote oral cancer. Two major therapeutic strategies may be adopted to keep GSK3β active. First and the most important will be to (**---**) prevent the inactivation of GSK3β, by targeting its upstream inhibitory kinases, so that they will remain unassociated. Second will be to (---) reconstitute the active GSK3β (Ala9GSK3β by gene therapy) to affected oral cancer sites.

The inhibition of GSK3β is regulated by various upstream kinases (PKA, PKB/Akt, PKC, p90RSK/MAPKAP, p70RS6K) [7,9,10,12,13,129]. PKA is predominantly controlled by extracellular signals (epidermal growth factor: EGF, platelet derived growth factor: PDGF), carcinogens and second messengers, mainly c-AMP. PKA activation in an OSCC cell line has been reported [63]. PKA-anchoring protein 220 (PKAP220) binds to both PKA and GSK3, bringing GSK3 into close proximity with PKA, which phosphorylates GSK3β to block its activity [130]. Recently, PKA has been identified as a therapeutic target in HNSCC; moreover, inhibition of PKA is known to affect many molecules (e.g., NFκB, Cyclin D1, Bcl-2, Cox-2 and p21), most of which are direct/indirect targets of GSK3β [131]. On the other hand, the activation of the PI3K/Akt pathway has been well studied in OSCC [15,127,132]. Direct evidence suggests that the pSer9GSK3β level in OSCC cell line is very high and can be decreased by inhibiting Akt signaling [62]. In addition, in oral cancer cells, blocking PI3K/Akt signaling causes more cells to undergo apoptosis; this effect is reversed by the use of a GSK3β inhibitor [63]. Akt signaling is important in HNSCC and is considered as a potential therapeutic target [133]. There is also evidence of PKC signaling in OSCC [11], and inhibition of PKC by pharmacological inhibitors reduces MMP-2 and MMP-9 [134], possibly via GSK3β. Suppression of PKC activity promotes GSK3β activity in epithelial cells, which increases apoptosis [7]. Targeting of PKCε has shown promising results in decreasing the invasion and mortality of HNSCC [135]. Moreover, p90RSK is known for its role in epithelial cell motility and invasiveness [136]. Tumor-promoting phorbol esters inhibit GSK3β via a classical MAPK cascade [19] by activating p90RSK (MAPKAP-KI). Therefore, the role of the p90RSK/GSK3β pathway might be important in oral cancer. Finally, GSK3β is inactivated by the mammalian target of rapamycin (mTOR) pathway, in which p70S6K phosphorylates GSK3β. In a SCC cell line, EGF inactivates GSK3β [137], which can be reversed by rapamycin at a concentration that blocks the activation of p70S6K [138]. Epidermal growth factor receptor (EGFR) activation in OSCC [137] might activate the p70S6K pathway [138]. Moreover, in HNSCC, p70S6K is reportedly very active, and targeting it with rapamycin has a potential anti-tumor effect *in vivo *[139], possibly due to the activation of GSK3β. All of these signaling pathways may have definite oncogenic properties and are activated by a variety of carcinogens or other cancer-promoting factors to induce oral cancer or cancers of similar epithelial origin. However, one thing that these oncogenic pathways share is that they all impinge on GSK3β inactivation. This may be the reason why, beyond geographical boundaries, all oral cancers are similar in their aggressiveness and their potential for migration and metastasis. Crosstalk is abundant in signal transduction pathways. Therefore, although targeting each of these pathways has a modest impact on oral cancer and causes toxicity to the patient, targeting GSK3β directly may be highly beneficial in treating OSCC [Fig. 4].

### Oral cancer etiology and intracellular signaling

The activation of established GSK3-inactivating upstream biological pathways by oral cancer-predisposing factors, such as tobacco, alcohol, and HPV, support the proposition of a causative role for GSK3β in OSCC. The role of carcinogens (from chewing and smoking tobacco) in oral cancer is firmly established [15,140]. Smokers show elevated levels of adenyle cyclase (AC) and PKA activity in oral epithelial cells [141,142]. Chewing areca nuts can lead to DNA damage and increased oxidative stress. The lime (calcium hydroxide) that coats the betel leaf promotes an alkaline oral environment, which activates Akt signaling [15]. There is accumulating evidence that connects nicotine-induced tumorigenesis to the activation of MAPK signaling [143], activation of PI3K/Akt signaling [144] and blocking of cytochrome *c*-mediated apoptosis [145]. Alcohol abuse increases the permeability of cells to carcinogens and activates PKA in cell culture [146]. HPV activates Akt in epithelial keratinocytes [4,147]. Moreover, a recent evaluation of epithelial tumors suggests that HPV infection can alter many biological pathways to maintain malignant processes by decreasing focal adhesion and up-regulating Wnt signaling and cell cycle genes [148]. Therefore, it is logical to hypothesize that the inactivation of GSK3β contributes to oral cancer.

### Evaluation of therapeutic potential and possible methods of targeting GSK3β in OSCC

Before selecting GSK3β as a therapeutic target in OSCC, its biological functions should be explored in detail. Though GSK3β has several isoforms, the isoform(s) specifically expressed in OSCC remain to be identified. If multiple isoforms are expressed, it will be important to understand their respective functions in oral cancer pathogenesis. The upstream cause of activation or inactivation of GSK3β as well as downstream target molecules and their status in OSCC should be thoroughly investigated at the patient level. Because it is an enzyme involved in regulating growth, cell cycle progression, apoptosis, and invasion, GSK3β may qualify as an ideal therapeutic target [123,149] for OSCC. Because of its role in both extrinsic and intrinsic apoptotic pathways, and because active GSK3β is nontoxic to non-cancerous cells (e.g., in a knock-in mouse study replacing Ser9 of GSK3β with Ala) [150], targeting the GSK3β pathway might be helpful in reducing unwanted apoptosis (in normal cells) and increasing useful apoptosis (in cancer cells).

The activation status of upstream molecules and the inactivation of GSK3β should be tested in different patients because each patient has a different lifestyle, etiological factors and genetic abnormalities. GSK3β can be inactivated by different upstream molecules in different oral tumors, even in the same patient. Inhibiting the upstream molecules pharmacologically by using peptide competitors and blocking phosphorylation at Ser9 certainly will keep GSK3β in an active state. The crystal structure of GSK3β peptide with an activated Akt ternary complex has been reported [151-154]. This may enable the design of small molecules that will disrupt the interaction of upstream kinases and GSK3β [Therapeutic strategy-I, Fig. 4] and thus prevent inhibitory kinases from associating with GSK3β. After checking the status of those patients who have inactivated GSK3β, Adenoviral vector carrying Ala9GSK3β may be tested along with other (chemo/radio) therapy, or with Ad-p53 (WT), which is known to block the progression of oral cancer to a certain extent [155]. However, although the chances are remote, some OSCC tumors will contain active GSK3β. It will be easy to test the inhibitors of GSK3 in these cases. The use of LiCl and SB-216763 in ovarian cancer [156]; LiCl and TDZD-8 in prostate cancer [157]; TDZD-8, SB-216763 and AR-A014418 in colorectal cancer [158,159]; LiCl, SB-216763, and TDZD-8 in myeloma [83]; TDZD-8 in AML and AML progenitor and stem cell cancer [160]; and LiCl and AR-A014418 in pancreatic cancer [161-163] has been evaluated, with positive outcomes. Almost all GSK3 inhibitors are able to inhibit two isoforms of GSK3 (α & β) with similar potency. The production and clinical evaluation of small-molecule inhibitors of particular isoforms will improve the chances of successful treatment in the future. Recent advancements in molecular biology have proven the effectiveness of small RNA interference (RNAi) in reducing the level of one protein by promoting mRNA degradation. This has been tried in an animal model of OSCC and as an alternative therapeutic strategy in patients who have developed drug resistance [164,165]. Similarly, RNAi has been used to counteract the overexpression of GSK3β in pancreatic [163], gastrointestinal [166], and prostate cancer [157], and it may be tried for OSCC.

## Conclusion

The goal of cancer drug discovery is to design non-toxic therapeutics that will be free of side effects. Thanks to a deepening understanding of cell biology and technological advancements, the concept of cancer therapy is being fine-tuned every day. Beginning with metabolic enzyme targeting using folate and methotrexate, to targeting of DNA polymerase and topoisomerase (tamoxifen), to selective hormonal targeting of estrogens/androgens via their nuclear hormone receptors, to the more recent advancement of targeting human growth factor receptor kinases and their effectors, the gradual improvements in our understanding of cancer biology have led to new and numerous therapeutics. Recent developments in molecular research have led to the hypothesis of "oncogene addiction," which suggest the continuous dependence of tumor cells on these oncogenes [167]. The inactivation of GSK3β in OSCC may behave like an oncogene, and its gradual/sustained inactivation may promote oral cancer. Though most of the upstream and downstream targets and their expression status correlate with the understanding of GSK3β inactivation, real, direct assessment should be attempted. If the activated form of GSK3β is non-toxic to normal oral epithelial cells, as was found in animal models [150], then the manipulation of the activated GSK3β provides hope for treating oral cancer. Unlike other molecules, GSK3β is one of the most attractive targets and is well understood because of extensive prior research on it. Therefore, it should be evaluated thoroughly as a potential target for the treatment of oral cancer.

## Competing interests

The authors declare that they have no competing interest.

## Authors' contributions

RM reviewed the literature, drafted and finalized the manuscript.

## References

[B1] Cheong SC Chandramouli GV Saleh A Zain RB Lau SH Sivakumaren S Pathmanathan R Prime SS Teo SH Patel V Gutkind JS Gene expression in human oral squamous cell carcinoma is influenced by risk factor exposure Oral Oncol 2009 45 712 719 10.1016/j.oraloncology.2008.11.002 19147396

[B2] Mishra A Bharti AC Saluja D Das BC Transactivation and expression patterns of Jun and Fos/AP-1 super-family proteins in human oral cancer Int J Cancer 2010 126 819 829 19653276 10.1002/ijc.24807

[B3] Scully C Bagan JV Recent advances in oral oncology 2008; squamous cell carcinoma imaging, treatment, prognostication and treatment outcomes Oral Oncol 2009 45 e25 30 10.1016/j.oraloncology.2008.12.011 19249236

[B4] zur Hausen H Papillomaviruses in the causation of human cancers - a brief historical account Virology 2009 384 260 265 10.1016/j.virol.2008.11.046 19135222

[B5] Paterson IC Eveson JW Prime SS Molecular changes in oral cancer may reflect aetiology and ethnic origin Eur J Cancer B Oral Oncol 1996 32B 150 153 10.1016/0964-1955(95)00065-8 8762870

[B6] Hamakawa H Nakashiro K Sumida T Shintani S Myers JN Takes RP Rinaldo A Ferlito A Basic evidence of molecular targeted therapy for oral cancer and salivary gland cancer Head Neck 2008 30 800 809 10.1002/hed.20830 18429007

[B7] Kim M Datta A Brakeman P Yu W Mostov KE Polarity proteins PAR6 and aPKC regulate cell death through GSK-3beta in 3D epithelial morphogenesis J Cell Sci 2007 120 2309 2317 10.1242/jcs.007443 17606986

[B8] Doble BW Woodgett JR GSK-3: tricks of the trade for a multi-tasking kinase J Cell Sci 2003 116 1175 1186 10.1242/jcs.00384 12615961 PMC3006448

[B9] Fang X Yu SX Lu Y Bast RC Jr Woodgett JR Mills GB Phosphorylation and inactivation of glycogen synthase kinase 3 by protein kinase A Proc Natl Acad Sci USA 2000 97 11960 11965 10.1073/pnas.220413597 11035810 PMC17277

[B10] Cross DA Alessi DR Cohen P Andjelkovich M Hemmings BA Inhibition of glycogen synthase kinase-3 by insulin mediated by protein kinase B Nature 1995 378 785 789 10.1038/378785a0 8524413

[B11] Kim MJ Lee JH Kim YK Myoung H Yun PY The role of tamoxifen in combination with cisplatin on oral squamous cell carcinoma cell lines Cancer Lett 2007 245 284 292 10.1016/j.canlet.2006.01.017 16513256

[B12] Stambolic V Woodgett JR Mitogen inactivation of glycogen synthase kinase-3 beta in intact cells via serine 9 phosphorylation Biochem J 1994 303 Pt 3 701 704 7980435 10.1042/bj3030701 PMC1137602

[B13] Sutherland C Leighton IA Cohen P Inactivation of glycogen synthase kinase-3 beta by phosphorylation: new kinase connections in insulin and growth-factor signalling Biochem J 1993 296 Pt 1 15 19 8250835 10.1042/bj2960015 PMC1137648

[B14] Lim J Kim JH Paeng JY Kim MJ Hong SD Lee JI Hong SP Prognostic value of activated Akt expression in oral squamous cell carcinoma J Clin Pathol 2005 58 1199 1205 10.1136/jcp.2004.024786 16254112 PMC1770780

[B15] Wu HT Ko SY Fong JH Chang KW Liu TY Kao SY Expression of phosphorylated Akt in oral carcinogenesis and its induction by nicotine and alkaline stimulation J Oral Pathol Med 2009 38 206 213 18331557 10.1111/j.1600-0714.2008.00659.x

[B16] Iamaroon A Krisanaprakornkit S Overexpression and activation of Akt2 protein in oral squamous cell carcinoma Oral Oncol 2009 45 e175 179 10.1016/j.oraloncology.2009.06.003 19628421

[B17] Embi N Rylatt DB Cohen P Glycogen synthase kinase-3 from rabbit skeletal muscle. Separation from cyclic-AMP-dependent protein kinase and phosphorylase kinase Eur J Biochem 1980 107 519 527 6249596

[B18] Woodgett JR Molecular cloning and expression of glycogen synthase kinase-3/factor A EMBO J 1990 9 2431 2438 2164470 10.1002/j.1460-2075.1990.tb07419.x PMC552268

[B19] Frame S Cohen P GSK3 takes centre stage more than 20 years after its discovery Biochem J 2001 359 1 16 10.1042/0264-6021:3590001 11563964 PMC1222116

[B20] Luo J Glycogen synthase kinase 3beta (GSK3beta) in tumorigenesis and cancer chemotherapy Cancer Lett 2009 273 194 200 10.1016/j.canlet.2008.05.045 18606491 PMC4978950

[B21] Manning G Whyte DB Martinez R Hunter T Sudarsanam S The protein kinase complement of the human genome Science 2002 298 1912 1934 10.1126/science.1075762 12471243

[B22] Darnell JE Jr Transcription factors as targets for cancer therapy Nat Rev Cancer 2002 2 740 749 10.1038/nrc906 12360277

[B23] Tsai WC Tsai ST Ko JY Jin YT Li C Huang W Young KC Lai MD Liu HS Wu LW The mRNA profile of genes in betel quid chewing oral cancer patients Oral Oncol 2004 40 418 426 10.1016/j.oraloncology.2003.09.015 14969821

[B24] de Groot RP Auwerx J Bourouis M Sassone-Corsi P Negative regulation of Jun/AP-1: conserved function of glycogen synthase kinase 3 and the Drosophila kinase shaggy Oncogene 1993 8 841 847 8384355

[B25] Buss H Dorrie A Schmitz ML Frank R Livingstone M Resch K Kracht M Phosphorylation of serine 468 by GSK-3beta negatively regulates basal p65 NF-kappaB activity J Biol Chem 2004 279 49571 49574 10.1074/jbc.C400442200 15465828

[B26] Yada M Hatakeyama S Kamura T Nishiyama M Tsunematsu R Imaki H Ishida N Okumura F Nakayama K Nakayama KI Phosphorylation-dependent degradation of c-Myc is mediated by the F-box protein Fbw7 EMBO J 2004 23 2116 2125 10.1038/sj.emboj.7600217 15103331 PMC424394

[B27] Ciani L Salinas PC WNTs in the vertebrate nervous system: from patterning to neuronal connectivity Nat Rev Neurosci 2005 6 351 362 10.1038/nrn1665 15832199

[B28] Yook JI Li XY Ota I Hu C Kim HS Kim NH Cha SY Ryu JK Choi YJ Kim J A Wnt-Axin2-GSK3beta cascade regulates Snail1 activity in breast cancer cells Nat Cell Biol 2006 8 1398 1406 10.1038/ncb1508 17072303

[B29] GA M Uddin S Mahmud D Damacela I Lavelle D Ahmed M van Besien K Wickrema A Regulation of myeloma cell growth through Akt/Gsk3/forkhead signaling pathway Biochem Biophys Res Commun 2002 297 760 764 10.1016/S0006-291X(02)02278-7 12359217

[B30] Ross SE Erickson RL Hemati N MacDougald OA Glycogen synthase kinase 3 is an insulin-regulated C/EBPalpha kinase Mol Cell Biol 1999 19 8433 8441 10567568 10.1128/mcb.19.12.8433 PMC84944

[B31] Gotschel F Kern C Lang S Sparna T Markmann C Schwager J McNelly S von Weizsacker F Laufer S Hecht A Merfort I Inhibition of GSK3 differentially modulates NF-kappaB, CREB, AP-1 and beta-catenin signaling in hepatocytes, but fails to promote TNF-alpha-induced apoptosis Exp Cell Res 2008 314 1351 1366 10.1016/j.yexcr.2007.12.015 18261723

[B32] de Sousa SO Mesquita RA Pinto DS Jr Gutkind S Immunolocalization of c-Fos and c-Jun in human oral mucosa and in oral squamous cell carcinoma J Oral Pathol Med 2002 31 78 81 11896827 10.1046/j.0904-2512.2001.10012.x

[B33] Pulverer BJ Kyriakis JM Avruch J Nikolakaki E Woodgett JR Phosphorylation of c-jun mediated by MAP kinases Nature 1991 353 670 674 10.1038/353670a0 1922387

[B34] Chen RH Juo PC Curran T Blenis J Phosphorylation of c-Fos at the C-terminus enhances its transforming activity Oncogene 1996 12 1493 1502 8622865

[B35] Mishra A Bharti AC Varghese P Saluja D Das BC Differential expression and activation of NF-kappaB family proteins during oral carcinogenesis: Role of high risk human papillomavirus infection Int J Cancer 2006 119 2840 2850 10.1002/ijc.22262 16998793

[B36] Sasahira T Kirita T Oue N Bhawal UK Yamamoto K Fujii K Ohmori H Luo Y Yasui W Bosserhoff AK Kuniyasu H High mobility group box-1-inducible melanoma inhibitory activity is associated with nodal metastasis and lymphangiogenesis in oral squamous cell carcinoma Cancer Sci 2008 99 1806 1812 18616526 10.1111/j.1349-7006.2008.00894.x PMC11159509

[B37] Ma Y Wang M Li N Wu R Wang X Bleomycin-induced nuclear factor-kappaB activation in human bronchial epithelial cells involves the phosphorylation of glycogen synthase kinase 3beta Toxicol Lett 2009 187 194 200 10.1016/j.toxlet.2009.02.023 19429264

[B38] Sanchez JF Sniderhan LF Williamson AL Fan S Chakraborty-Sett S Maggirwar SB Glycogen synthase kinase 3beta-mediated apoptosis of primary cortical astrocytes involves inhibition of nuclear factor kappaB signaling Mol Cell Biol 2003 23 4649 4662 10.1128/MCB.23.13.4649-4662.2003 12808104 PMC164840

[B39] Bachelder RE Yoon SO Franci C de Herreros AG Mercurio AM Glycogen synthase kinase-3 is an endogenous inhibitor of Snail transcription: implications for the epithelial-mesenchymal transition J Cell Biol 2005 168 29 33 10.1083/jcb.200409067 15631989 PMC2171685

[B40] Tian D Zhu M Chen WS Li JS Wu RL Wang X Role of glycogen synthase kinase 3 in squamous differentiation induced by cigarette smoke in porcine tracheobronchial epithelial cells Food Chem Toxicol 2006 44 1590 1596 10.1016/j.fct.2006.03.013 16750592

[B41] Vora HH Shah NG Trivedi TI Goswami JV Shukla SN Shah PM Expression of C-Myc mRNA in squamous cell carcinoma of the tongue J Surg Oncol 2007 95 70 78 10.1002/jso.20675 17192869

[B42] Baral R Patnaik S Das BR Co-overexpression of p53 and c-myc proteins linked with advanced stages of betel- and tobacco-related oral squamous cell carcinomas from eastern India Eur J Oral Sci 1998 106 907 913 10.1046/j.0909-8836.1998.eos106502.x 9786319

[B43] Mahomed F Altini M Meer S Altered E-cadherin/beta-catenin expression in oral squamous carcinoma with and without nodal metastasis Oral Dis 2007 13 386 392 10.1111/j.1601-0825.2006.01295.x 17577324

[B44] Tanaka N Odajima T Ogi K Ikeda T Satoh M Expression of E-cadherin, alpha-catenin, and beta-catenin in the process of lymph node metastasis in oral squamous cell carcinoma Br J Cancer 2003 89 557 563 10.1038/sj.bjc.6601124 12888830 PMC2394393

[B45] Bankfalvi A Krassort M Vegh A Felszeghy E Piffko J Deranged expression of the E-cadherin/beta-catenin complex and the epidermal growth factor receptor in the clinical evolution and progression of oral squamous cell carcinomas J Oral Pathol Med 2002 31 450 457 10.1034/j.1600-0714.2002.00147.x 12220351

[B46] de Castro J Gamallo C Palacios J Moreno-Bueno G Rodriguez N Feliu J Gonzalez-Baron M beta-catenin expression pattern in primary oesophageal squamous cell carcinoma. Relationship with clinicopathologic features and clinical outcome Virchows Arch 2000 437 599 604 10.1007/s004280000266 11193470

[B47] Bahram F von der Lehr N Cetinkaya C Larsson LG c-Myc hot spot mutations in lymphomas result in inefficient ubiquitination and decreased proteasome-mediated turnover Blood 2000 95 2104 2110 10706881

[B48] Oster SK Ho CS Soucie EL Penn LZ The myc oncogene: MarvelouslY Complex Adv Cancer Res 2002 84 81 154 full_text 11885563 10.1016/s0065-230x(02)84004-0

[B49] Gregory MA Qi Y Hann SR Phosphorylation by glycogen synthase kinase-3 controls c-myc proteolysis and subnuclear localization J Biol Chem 2003 278 51606 51612 10.1074/jbc.M310722200 14563837

[B50] An J Yang DY Xu QZ Zhang SM Huo YY Shang ZF Wang Y Wu DC Zhou PK DNA-dependent protein kinase catalytic subunit modulates the stability of c-Myc oncoprotein Mol Cancer 2008 7 32 10.1186/1476-4598-7-32 18426604 PMC2383926

[B51] Sogabe Y Suzuki H Toyota M Ogi K Imai T Nojima M Sasaki Y Hiratsuka H Tokino T Epigenetic inactivation of SFRP genes in oral squamous cell carcinoma Int J Oncol 2008 32 1253 1261 18497987 10.3892/ijo_32_6_1253

[B52] Iwai S Katagiri W Kong C Amekawa S Nakazawa M Yura Y Mutations of the APC, beta-catenin, and axin 1 genes and cytoplasmic accumulation of beta-catenin in oral squamous cell carcinoma J Cancer Res Clin Oncol 2005 131 773 782 10.1007/s00432-005-0027-y 16163548 PMC12161198

[B53] Yeh KT Chang JG Lin TH Wang YF Chang JY Shih MC Lin CC Correlation between protein expression and epigenetic and mutation changes of Wnt pathway-related genes in oral cancer Int J Oncol 2003 23 1001 1007 12963979

[B54] Franz M Spiegel K Umbreit C Richter P Codina-Canet C Berndt A Altendorf-Hofmann A Koscielny S Hyckel P Kosmehl H Virtanen I Expression of Snail is associated with myofibroblast phenotype development in oral squamous cell carcinoma Histochem Cell Biol 2009 131 651 660 10.1007/s00418-009-0559-3 19198871

[B55] Zhou BP Deng J Xia W Xu J Li YM Gunduz M Hung MC Dual regulation of Snail by GSK-3beta-mediated phosphorylation in control of epithelial-mesenchymal transition Nat Cell Biol 2004 6 931 940 10.1038/ncb1173 15448698

[B56] Doble BW Woodgett JR Role of glycogen synthase kinase-3 in cell fate and epithelial-mesenchymal transitions Cells Tissues Organs 2007 185 73 84 10.1159/000101306 17587811

[B57] Oren M Decision making by p53: life, death and cancer Cell Death Differ 2003 10 431 442 10.1038/sj.cdd.4401183 12719720

[B58] Watcharasit P Bijur GN Song L Zhu J Chen X Jope RS Glycogen synthase kinase-3beta (GSK3beta) binds to and promotes the actions of p53 J Biol Chem 2003 278 48872 48879 10.1074/jbc.M305870200 14523002 PMC1361697

[B59] Eom TY Jope RS GSK3 beta N-terminus binding to p53 promotes its acetylation Mol Cancer 2009 8 14 10.1186/1476-4598-8-14 19265551 PMC2660897

[B60] Ma C Wang J Gao Y Gao TW Chen G Bower KA Odetallah M Ding M Ke Z Luo J The role of glycogen synthase kinase 3beta in the transformation of epidermal cells Cancer Res 2007 67 7756 7764 10.1158/0008-5472.CAN-06-4665 17699780

[B61] Chun KH Lee HY Hassan K Khuri F Hong WK Lotan R Implication of protein kinase B/Akt and Bcl-2/Bcl-XL suppression by the farnesyl transferase inhibitor SCH66336 in apoptosis induction in squamous carcinoma cells Cancer Res 2003 63 4796 4800 12941797

[B62] Amornphimoltham P Sriuranpong V Patel V Benavides F Conti CJ Sauk J Sausville EA Molinolo AA Gutkind JS Persistent activation of the Akt pathway in head and neck squamous cell carcinoma: a potential target for UCN-01 Clin Cancer Res 2004 10 4029 4037 10.1158/1078-0432.CCR-03-0249 15217935

[B63] Suzuki M Shinohara F Endo M Sugazaki M Echigo S Rikiishi H Zebularine suppresses the apoptotic potential of 5-fluorouracil via cAMP/PKA/CREB pathway against human oral squamous cell carcinoma cells Cancer Chemother Pharmacol 2009 64 223 232 10.1007/s00280-008-0833-4 18830594

[B64] Bauer K Dowejko A Bosserhoff AK Reichert TE Bauer RJ P-cadherin induces an epithelial-like phenotype in oral squamous cell carcinoma by GSK-3beta-mediated Snail phosphorylation Carcinogenesis 2009 30 1781 1788 10.1093/carcin/bgp175 19654099

[B65] Malumbres M Barbacid M Cell cycle, CDKs and cancer: a changing paradigm Nat Rev Cancer 2009 9 153 166 10.1038/nrc2602 19238148

[B66] Sartor M Steingrimsdottir H Elamin F Gaken J Warnakulasuriya S Partridge M Thakker N Johnson NW Tavassoli M Role of p16/MTS1, cyclin D1 and RB in primary oral cancer and oral cancer cell lines Br J Cancer 1999 80 79 86 10.1038/sj.bjc.6690505 10389982 PMC2363027

[B67] Turatti E da Costa Neves A de Magalhaes MH de Sousa SO Assessment of c-Jun, c-Fos and cyclin D1 in premalignant and malignant oral lesions J Oral Sci 2005 47 71 76 10.2334/josnusd.47.71 16050486

[B68] Mihara M Shintani S Nakahara Y Kiyota A Ueyama Y Matsumura T Wong DT Overexpression of CDK2 is a prognostic indicator of oral cancer progression Jpn J Cancer Res 2001 92 352 360 11267947 10.1111/j.1349-7006.2001.tb01102.x PMC5926707

[B69] Fraczek M Wozniak Z Ramsey D Krecicki T Expression patterns of cyclin E, cyclin A and CDC25 phosphatases in laryngeal carcinogenesis Eur Arch Otorhinolaryngol 2007 264 923 928 10.1007/s00405-007-0276-2 17361412

[B70] Diehl JA Cheng M Roussel MF Sherr CJ Glycogen synthase kinase-3beta regulates cyclin D1 proteolysis and subcellular localization Genes Dev 1998 12 3499 3511 10.1101/gad.12.22.3499 9832503 PMC317244

[B71] Welcker M Singer J Loeb KR Grim J Bloecher A Gurien-West M Clurman BE Roberts JM Multisite phosphorylation by Cdk2 and GSK3 controls cyclin E degradation Mol Cell 2003 12 381 392 10.1016/S1097-2765(03)00287-9 14536078

[B72] Leis H Segrelles C Ruiz S Santos M Paramio JM Expression, localization, and activity of glycogen synthase kinase 3beta during mouse skin tumorigenesis Mol Carcinog 2002 35 180 185 10.1002/mc.10087 12489109

[B73] Goto H Kawano K Kobayashi I Sakai H Yanagisawa S Expression of cyclin D1 and GSK-3beta and their predictive value of prognosis in squamous cell carcinomas of the tongue Oral Oncol 2002 38 549 556 10.1016/S1368-8375(01)00121-X 12167432

[B74] Tokumaru Y Yamashita K Osada M Nomoto S Sun DI Xiao Y Hoque MO Westra WH Califano JA Sidransky D Inverse correlation between cyclin A1 hypermethylation and p53 mutation in head and neck cancer identified by reversal of epigenetic silencing Cancer Res 2004 64 5982 5987 10.1158/0008-5472.CAN-04-0993 15342377

[B75] Yamazaki K Hasegawa M Ohoka I Hanami K Asoh A Nagao T Sugano I Ishida Y Increased E2F-1 expression via tumour cell proliferation and decreased apoptosis are correlated with adverse prognosis in patients with squamous cell carcinoma of the oesophagus J Clin Pathol 2005 58 904 910 10.1136/jcp.2004.023127 16126868 PMC1770838

[B76] Meyer N Penn LZ Reflecting on 25 years with MYC Nat Rev Cancer 2008 8 976 990 10.1038/nrc2231 19029958

[B77] Miliani de Marval PL Macias E Rounbehler R Sicinski P Kiyokawa H Johnson DG Conti CJ Rodriguez-Puebla ML Lack of cyclin-dependent kinase 4 inhibits c-myc tumorigenic activities in epithelial tissues Mol Cell Biol 2004 24 7538 7547 10.1128/MCB.24.17.7538-7547.2004 15314163 PMC506988

[B78] Nadal A Jares P Pinyol M Conde L Romeu C Fernandez PL Campo E Cardesa A Association of CDK4 and CCND1 mRNA overexpression in laryngeal squamous cell carcinomas occurs without CDK4 amplification Virchows Arch 2007 450 161 167 10.1007/s00428-006-0314-2 17139501

[B79] Nemes JA Nemes Z Marton IJ p21WAF1/CIP1 expression is a marker of poor prognosis in oral squamous cell carcinoma J Oral Pathol Med 2005 34 274 279 10.1111/j.1600-0714.2005.00310.x 15817070

[B80] Yokoyama K Kamata N Fujimoto R Tsutsumi S Tomonari M Taki M Hosokawa H Nagayama M Increased invasion and matrix metalloproteinase-2 expression by Snail-induced mesenchymal transition in squamous cell carcinomas Int J Oncol 2003 22 891 898 12632084

[B81] Rossig L Badorff C Holzmann Y Zeiher AM Dimmeler S Glycogen synthase kinase-3 couples AKT-dependent signaling to the regulation of p21Cip1 degradation J Biol Chem 2002 277 9684 9689 10.1074/jbc.M106157200 11779850

[B82] Kudo Y Kitajima S Ogawa I Miyauchi M Takata T Down-regulation of Cdk inhibitor p27 in oral squamous cell carcinoma Oral Oncol 2005 41 105 116 10.1016/j.oraloncology.2004.05.003 15695111

[B83] Zhou Y Uddin S Zimmerman T Kang JA Ulaszek J Wickrema A Growth control of multiple myeloma cells through inhibition of glycogen synthase kinase-3 Leuk Lymphoma 2008 49 1945 1953 10.1080/10428190802304966 18728964 PMC2574790

[B84] Reagan-Shaw S Ahmad N RNA interference-mediated depletion of phosphoinositide 3-kinase activates forkhead box class O transcription factors and induces cell cycle arrest and apoptosis in breast carcinoma cells Cancer Res 2006 66 1062 1069 10.1158/0008-5472.CAN-05-1018 16424042

[B85] Ying J Srivastava G Hsieh WS Gao Z Murray P Liao SK Ambinder R Tao Q The stress-responsive gene GADD45G is a functional tumor suppressor, with its response to environmental stresses frequently disrupted epigenetically in multiple tumors Clin Cancer Res 2005 11 6442 6449 10.1158/1078-0432.CCR-05-0267 16166418

[B86] Chen JC Lu KW Tsai ML Hsu SC Kuo CL Yang JS Hsia TC Yu CS Chou ST Kao MC Gypenosides induced G0/G1 arrest via CHk2 and apoptosis through endoplasmic reticulum stress and mitochondria-dependent pathways in human tongue cancer SCC-4 cells Oral Oncol 2009 45 273 283 10.1016/j.oraloncology.2008.05.012 18674953

[B87] Obaya AJ Mateyak MK Sedivy JM Mysterious liaisons: the relationship between c-Myc and the cell cycle Oncogene 1999 18 2934 2941 10.1038/sj.onc.1202749 10378690

[B88] Kang T Wei Y Honaker Y Yamaguchi H Appella E Hung MC Piwnica-Worms H GSK-3 beta targets Cdc25A for ubiquitin-mediated proteolysis, and GSK-3 beta inactivation correlates with Cdc25A overproduction in human cancers Cancer Cell 2008 13 36 47 10.1016/j.ccr.2007.12.002 18167338 PMC2276649

[B89] Rosivatz E Becker I Specht K Fricke E Luber B Busch R Hofler H Becker KF Differential expression of the epithelial-mesenchymal transition regulators snail, SIP1, and twist in gastric cancer Am J Pathol 2002 161 1881 1891 12414534 10.1016/S0002-9440(10)64464-1 PMC1850763

[B90] Yang MH Chang SY Chiou SH Liu CJ Chi CW Chen PM Teng SC Wu KJ Overexpression of NBS1 induces epithelial-mesenchymal transition and co-expression of NBS1 and Snail predicts metastasis of head and neck cancer Oncogene 2007 26 1459 1467 10.1038/sj.onc.1209929 16936774

[B91] Kosmehl H Berndt A Strassburger S Borsi L Rousselle P Mandel U Hyckel P Zardi L Katenkamp D Distribution of laminin and fibronectin isoforms in oral mucosa and oral squamous cell carcinoma Br J Cancer 1999 81 1071 1079 10.1038/sj.bjc.6690809 10576667 PMC2362955

[B92] Mhawech P Dulguerov P Assaly M Ares C Allal AS EB-D fibronectin expression in squamous cell carcinoma of the head and neck Oral Oncol 2005 41 82 88 10.1016/j.oraloncology.2004.07.003 15598590

[B93] de Nigris F Botti C Rossiello R Crimi E Sica V Napoli C Cooperation between Myc and YY1 provides novel silencing transcriptional targets of alpha3beta1-integrin in tumour cells Oncogene 2007 26 382 394 10.1038/sj.onc.1209804 16878156

[B94] Haraguchi M Okubo T Miyashita Y Miyamoto Y Hayashi M Crotti TN McHugh KP Ozawa M Snail regulates cell-matrix adhesion by regulation of the expression of integrins and basement membrane proteins J Biol Chem 2008 283 23514 23523 10.1074/jbc.M801125200 18593711 PMC3259798

[B95] Erdem NF Carlson ER Gerard DA Ichiki AT Characterization of 3 oral squamous cell carcinoma cell lines with different invasion and/or metastatic potentials J Oral Maxillofac Surg 2007 65 1725 1733 10.1016/j.joms.2006.11.034 17719389

[B96] Ziober BL Silverman SS Jr Kramer RH Adhesive mechanisms regulating invasion and metastasis in oral cancer Crit Rev Oral Biol Med 2001 12 499 510 10.1177/10454411010120060401 11806519

[B97] Kornberg LJ Focal adhesion kinase expression in oral cancers Head Neck 1998 20 634 639 10.1002/(SICI)1097-0347(199810)20:7<634::AID-HED10>3.0.CO;2-M 9744465

[B98] Ko BS Chang TC Chen CH Liu CC Kuo CC Hsu C Shen YC Shen TL Golubovskaya VM Chang CC Bortezomib suppresses focal adhesion kinase expression via interrupting nuclear factor-kappa B Life Sci 2010 86 199 206 10.1016/j.lfs.2009.12.003 20006625

[B99] Bianchi M De Lucchini S Marin O Turner DL Hanks SK Villa-Moruzzi E Regulation of FAK Ser-722 phosphorylation and kinase activity by GSK3 and PP1 during cell spreading and migration Biochem J 2005 391 359 370 10.1042/BJ20050282 15975092 PMC1276935

[B100] Ramos DM Dang D Sadler S The role of the integrin alpha v beta6 in regulating the epithelial to mesenchymal transition in oral cancer Anticancer Res 2009 29 125 130 19331141

[B101] Han S Roman J COX-2 inhibitors suppress integrin alpha5 expression in human lung carcinoma cells through activation of Erk: involvement of Sp1 and AP-1 sites Int J Cancer 2005 116 536 546 10.1002/ijc.21125 15825163

[B102] Zutter MM Santoro SA Painter AS Tsung YL Gafford A The human alpha 2 integrin gene promoter. Identification of positive and negative regulatory elements important for cell-type and developmentally restricted gene expression J Biol Chem 1994 269 463 469 8276836

[B103] Nishida K Kitazawa R Mizuno K Maeda S Kitazawa S Identification of regulatory elements of human alpha 6 integrin subunit gene Biochem Biophys Res Commun 1997 241 258 263 10.1006/bbrc.1997.7808 9425259

[B104] Corbi AL Jensen UB Watt FM The alpha2 and alpha5 integrin genes: identification of transcription factors that regulate promoter activity in epidermal keratinocytes FEBS Lett 2000 474 201 207 10.1016/S0014-5793(00)01591-X 10838085

[B105] Barrallo-Gimeno A Nieto MA The Snail genes as inducers of cell movement and survival: implications in development and cancer Development 2005 132 3151 3161 10.1242/dev.01907 15983400

[B106] Jordan RC Macabeo-Ong M Shiboski CH Dekker N Ginzinger DG Wong DT Schmidt BL Overexpression of matrix metalloproteinase-1 and -9 mRNA is associated with progression of oral dysplasia to cancer Clin Cancer Res 2004 10 6460 6465 10.1158/1078-0432.CCR-04-0656 15475433

[B107] Katayama A Bandoh N Kishibe K Takahara M Ogino T Nonaka S Harabuchi Y Expressions of matrix metalloproteinases in early-stage oral squamous cell carcinoma as predictive indicators for tumor metastases and prognosis Clin Cancer Res 2004 10 634 640 10.1158/1078-0432.CCR-0864-02 14760086

[B108] Sutinen M Kainulainen T Hurskainen T Vesterlund E Alexander JP Overall CM Sorsa T Salo T Expression of matrix metalloproteinases (MMP-1 and -2) and their inhibitors (TIMP-1, -2 and -3) in oral lichen planus, dysplasia, squamous cell carcinoma and lymph node metastasis Br J Cancer 1998 77 2239 2245 9649139 10.1038/bjc.1998.372 PMC2150416

[B109] Impola U Uitto VJ Hietanen J Hakkinen L Zhang L Larjava H Isaka K Saarialho-Kere U Differential expression of matrilysin-1 (MMP-7), 92 kD gelatinase (MMP-9), and metalloelastase (MMP-12) in oral verrucous and squamous cell cancer J Pathol 2004 202 14 22 10.1002/path.1479 14694517

[B110] Kinugasa Y Hatori M Ito H Kurihara Y Ito D Nagumo M Inhibition of cyclooxygenase-2 suppresses invasiveness of oral squamous cell carcinoma cell lines via down-regulation of matrix metalloproteinase-2 and CD44 Clin Exp Metastasis 2004 21 737 745 10.1007/s10585-005-1190-x 16035618

[B111] Kosunen A Pirinen R Ropponen K Pukkila M Kellokoski J Virtaniemi J Sironen R Juhola M Kumpulainen E Johansson R CD44 expression and its relationship with MMP-9, clinicopathological factors and survival in oral squamous cell carcinoma Oral Oncol 2007 43 51 59 10.1016/j.oraloncology.2006.01.003 16798062

[B112] Lee CH Liu SY Lin MH Chiang WF Chen TC Huang WT Chou DS Chiu CT Liu YC Upregulation of matrix metalloproteinase-1 (MMP-1) expression in oral carcinomas of betel quid (BQ) users: roles of BQ ingredients in the acceleration of tumour cell motility through MMP-1 Arch Oral Biol 2008 53 810 818 10.1016/j.archoralbio.2008.05.004 18571622

[B113] Yokoyama K Kamata N Hayashi E Hoteiya T Ueda N Fujimoto R Nagayama M Reverse correlation of E-cadherin and snail expression in oral squamous cell carcinoma cells in vitro Oral Oncol 2001 37 65 71 10.1016/S1368-8375(00)00059-2 11120485

[B114] Taki M Kamata N Yokoyama K Fujimoto R Tsutsumi S Nagayama M Down-regulation of Wnt-4 and up-regulation of Wnt-5a expression by epithelial-mesenchymal transition in human squamous carcinoma cells Cancer Sci 2003 94 593 597 10.1111/j.1349-7006.2003.tb01488.x 12841867 PMC11160266

[B115] Schipper JH Frixen UH Behrens J Unger A Jahnke K Birchmeier W E-cadherin expression in squamous cell carcinomas of head and neck: inverse correlation with tumor dedifferentiation and lymph node metastasis Cancer Res 1991 51 6328 6337 1933895

[B116] Batlle E Sancho E Franci C Dominguez D Monfar M Baulida J Garcia De Herreros A The transcription factor snail is a repressor of E-cadherin gene expression in epithelial tumour cells Nat Cell Biol 2000 2 84 89 10.1038/35000034 10655587

[B117] Sappayatosok K Maneerat Y Swasdison S Viriyavejakul P Dhanuthai K Zwang J Chaisri U Expression of pro-inflammatory protein, iNOS, VEGF and COX-2 in oral squamous cell carcinoma (OSCC), relationship with angiogenesis and their clinico-pathological correlation Med Oral Patol Oral Cir Bucal 2009 14 E319 324 19300368

[B118] Nystrom ML McCulloch D Weinreb PH Violette SM Speight PM Marshall JF Hart IR Thomas GJ Cyclooxygenase-2 inhibition suppresses alphavbeta6 integrin-dependent oral squamous carcinoma invasion Cancer Res 2006 66 10833 10842 10.1158/0008-5472.CAN-06-1640 17108119

[B119] Kurihara Y Hatori M Ando Y Ito D Toyoshima T Tanaka M Shintani S Inhibition of cyclooxygenase-2 suppresses the invasiveness of oral squamous cell carcinoma cell lines via down-regulation of matrix metalloproteinase-2 production and activation Clin Exp Metastasis 2009 26 425 432 10.1007/s10585-009-9241-3 19241124

[B120] Subbaramaiah K Altorki N Chung WJ Mestre JR Sampat A Dannenberg AJ Inhibition of cyclooxygenase-2 gene expression by p53 J Biol Chem 1999 274 10911 10915 10.1074/jbc.274.16.10911 10196169

[B121] Chiang SL Chen PH Lee CH Ko AM Lee KW Lin YC Ho PS Tu HP Wu DC Shieh TY Ko YC Up-regulation of inflammatory signalings by areca nut extract and role of cyclooxygenase-2 -1195G > a polymorphism reveal risk of oral cancer Cancer Res 2008 68 8489 8498 10.1158/0008-5472.CAN-08-0823 18922923

[B122] Tamatani T Azuma M Ashida Y Motegi K Takashima R Harada K Kawaguchi S Sato M Enhanced radiosensitization and chemosensitization in NF-kappaB-suppressed human oral cancer cells via the inhibition of gamma-irradiation- and 5-FU-induced production of IL-6 and IL-8 Int J Cancer 2004 108 912 921 10.1002/ijc.11640 14712497

[B123] Beurel E Jope RS The paradoxical pro- and anti-apoptotic actions of GSK3 in the intrinsic and extrinsic apoptosis signaling pathways Prog Neurobiol 2006 79 173 189 10.1016/j.pneurobio.2006.07.006 16935409 PMC1618798

[B124] Hoeflich KP Luo J Rubie EA Tsao MS Jin O Woodgett JR Requirement for glycogen synthase kinase-3beta in cell survival and NF-kappaB activation Nature 2000 406 86 90 10.1038/35017574 10894547

[B125] Tan J Zhuang L Leong HS Iyer NG Liu ET Yu Q Pharmacologic modulation of glycogen synthase kinase-3beta promotes p53-dependent apoptosis through a direct Bax-mediated mitochondrial pathway in colorectal cancer cells Cancer Res 2005 65 9012 9020 10.1158/0008-5472.CAN-05-1226 16204075

[B126] Lonze BE Ginty DD Function and regulation of CREB family transcription factors in the nervous system Neuron 2002 35 605 623 10.1016/S0896-6273(02)00828-0 12194863

[B127] Uchida M Iwase M Takaoka S Yoshiba S Kondo G Watanabe H Ohashi M Nagumo M Shintani S Enhanced susceptibility to tumor necrosis factor-related apoptosis-inducing ligand-mediated apoptosis in oral squamous cell carcinoma cells treated with phosphatidylinositol 3-kinase inhibitors Int J Oncol 2007 30 1163 1171 17390018

[B128] Belkhiri A Dar AA Zaika A Kelley M El-Rifai W t-Darpp promotes cancer cell survival by up-regulation of Bcl2 through Akt-dependent mechanism Cancer Res 2008 68 395 403 10.1158/0008-5472.CAN-07-1580 18199533

[B129] Ballou LM Tian PY Lin HY Jiang YP Lin RZ Dual regulation of glycogen synthase kinase-3beta by the alpha1A-adrenergic receptor J Biol Chem 2001 276 40910 40916 10.1074/jbc.M103480200 11533051

[B130] Tanji C Yamamoto H Yorioka N Kohno N Kikuchi K Kikuchi A A-kinase anchoring protein AKAP220 binds to glycogen synthase kinase-3beta (GSK-3beta) and mediates protein kinase A-dependent inhibition of GSK-3beta J Biol Chem 2002 277 36955 36961 10.1074/jbc.M206210200 12147701

[B131] Arun P Brown MS Ehsanian R Chen Z Van Waes C Nuclear NF-kappaB p65 phosphorylation at serine 276 by protein kinase A contributes to the malignant phenotype of head and neck cancer Clin Cancer Res 2009 15 5974 5984 10.1158/1078-0432.CCR-09-1352 19789307 PMC2760015

[B132] Tosi L Rinaldi E Carinci F Farina A Pastore A Pelucchi S Cassano L Evangelisti R Carinci P Volinia S Akt, protein kinase C, and mitogen-activated protein kinase phosphorylation status in head and neck squamous cell carcinoma Head Neck 2005 27 130 137 10.1002/hed.20120 15641106

[B133] Moral M Paramio JM Akt pathway as a target for therapeutic intervention in HNSCC Histol Histopathol 2008 23 1269 1278 18712679 10.14670/HH-23.1269

[B134] Tsai CH Hsieh YS Yang SF Chou MY Chang YC Matrix metalloproteinase 2 and matrix metalloproteinase 9 expression in human oral squamous cell carcinoma and the effect of protein kinase C inhibitors: preliminary observations Oral Surg Oral Med Oral Pathol Oral Radiol Endod 2003 95 710 716 10.1067/moe.2003.121 12789153

[B135] Pan Q Bao LW Teknos TN Merajver SD Targeted disruption of protein kinase C epsilon reduces cell invasion and motility through inactivation of RhoA and RhoC GTPases in head and neck squamous cell carcinoma Cancer Res 2006 66 9379 9384 10.1158/0008-5472.CAN-06-2646 17018591 PMC4383316

[B136] Doehn U Hauge C Frank SR Jensen CJ Duda K Nielsen JV Cohen MS Johansen JV Winther BR Lund LR RSK is a principal effector of the RAS-ERK pathway for eliciting a coordinate promotile/invasive gene program and phenotype in epithelial cells Mol Cell 2009 35 511 522 10.1016/j.molcel.2009.08.002 19716794 PMC3784321

[B137] Sheu JJ Hua CH Wan L Lin YJ Lai MT Tseng HC Jinawath N Tsai MH Chang NW Lin CF Functional genomic analysis identified epidermal growth factor receptor activation as the most common genetic event in oral squamous cell carcinoma Cancer Res 2009 69 2568 2576 10.1158/0008-5472.CAN-08-3199 19276369

[B138] Saito Y Vandenheede JR Cohen P The mechanism by which epidermal growth factor inhibits glycogen synthase kinase 3 in A431 cells Biochem J 1994 303 Pt 1 27 31 7945252 10.1042/bj3030027 PMC1137551

[B139] Amornphimoltham P Patel V Sodhi A Nikitakis NG Sauk JJ Sausville EA Molinolo AA Gutkind JS Mammalian target of rapamycin, a molecular target in squamous cell carcinomas of the head and neck Cancer Res 2005 65 9953 9961 10.1158/0008-5472.CAN-05-0921 16267020

[B140] West KA Brognard J Clark AS Linnoila IR Yang X Swain SM Harris C Belinsky S Dennis PA Rapid Akt activation by nicotine and a tobacco carcinogen modulates the phenotype of normal human airway epithelial cells J Clin Invest 2003 111 81 90 12511591 10.1172/JCI16147 PMC151834

[B141] Hope BT Nagarkar D Leonard S Wise RA Long-term upregulation of protein kinase A and adenylate cyclase levels in human smokers J Neurosci 2007 27 1964 1972 10.1523/JNEUROSCI.3661-06.2007 17314292 PMC2575739

[B142] Du B Altorki NK Kopelovich L Subbaramaiah K Dannenberg AJ Tobacco smoke stimulates the transcription of amphiregulin in human oral epithelial cells: evidence of a cyclic AMP-responsive element binding protein-dependent mechanism Cancer Res 2005 65 5982 5988 10.1158/0008-5472.CAN-05-0628 15994978

[B143] Hecht SS Cigarette smoking and lung cancer: chemical mechanisms and approaches to prevention Lancet Oncol 2002 3 461 469 10.1016/S1470-2045(02)00815-X 12147432

[B144] Nakayama H Numakawa T Ikeuchi T Nicotine-induced phosphorylation of Akt through epidermal growth factor receptor and Src in PC12h cells J Neurochem 2002 83 1372 1379 10.1046/j.1471-4159.2002.01248.x 12472891

[B145] Sugano N Minegishi T Kawamoto K Ito K Nicotine inhibits UV-induced activation of the apoptotic pathway Toxicol Lett 2001 125 61 65 10.1016/S0378-4274(01)00416-7 11701223

[B146] Etique N Flament S Lecomte J Grillier-Vuissoz I Ethanol-induced ligand-independent activation of ERalpha mediated by cyclic AMP/PKA signaling pathway: an in vitro study on MCF-7 breast cancer cells Int J Oncol 2007 31 1509 1518 17982678

[B147] Pim D Massimi P Dilworth SM Banks L Activation of the protein kinase B pathway by the HPV-16 E7 oncoprotein occurs through a mechanism involving interaction with PP2A Oncogene 2005 24 7830 7838 10.1038/sj.onc.1208935 16044149

[B148] Perez-Plasencia C Vazquez-Ortiz G Lopez-Romero R Pina-Sanchez P Moreno J Salcedo M Genome wide expression analysis in HPV16 Cervical Cancer: identification of altered metabolic pathways Infect Agent Cancer 2007 2 16 10.1186/1750-9378-2-16 17822553 PMC2034543

[B149] Sun Y p53 and its downstream proteins as molecular targets of cancer Mol Carcinog 2006 45 409 415 10.1002/mc.20231 16652354

[B150] Matsuda T Zhai P Maejima Y Hong C Gao S Tian B Goto K Takagi H Tamamori-Adachi M Kitajima S Sadoshima J Distinct roles of GSK-3alpha and GSK-3beta phosphorylation in the heart under pressure overload Proc Natl Acad Sci USA 2008 105 20900 20905 10.1073/pnas.0808315106 19106302 PMC2634936

[B151] Yang J Cron P Good VM Thompson V Hemmings BA Barford D Crystal structure of an activated Akt/protein kinase B ternary complex with GSK3-peptide and AMP-PNP Nat Struct Biol 2002 9 940 944 10.1038/nsb870 12434148

[B152] Dajani R Fraser E Roe SM Yeo M Good VM Thompson V Dale TC Pearl LH Structural basis for recruitment of glycogen synthase kinase 3beta to the axin-APC scaffold complex EMBO J 2003 22 494 501 10.1093/emboj/cdg068 12554650 PMC140752

[B153] Bax B Carter PS Lewis C Guy AR Bridges A Tanner R Pettman G Mannix C Culbert AA Brown MJ The structure of phosphorylated GSK-3beta complexed with a peptide, FRATtide, that inhibits beta-catenin phosphorylation Structure 2001 9 1143 1152 10.1016/S0969-2126(01)00679-7 11738041

[B154] Dajani R Fraser E Roe SM Young N Good V Dale TC Pearl LH Crystal structure of glycogen synthase kinase 3 beta: structural basis for phosphate-primed substrate specificity and autoinhibition Cell 2001 105 721 732 10.1016/S0092-8674(01)00374-9 11440715

[B155] Clayman GL el-Naggar AK Lippman SM Henderson YC Frederick M Merritt JA Zumstein LA Timmons TM Liu TJ Ginsberg L Adenovirus-mediated p53 gene transfer in patients with advanced recurrent head and neck squamous cell carcinoma J Clin Oncol 1998 16 2221 2232 9626224 10.1200/JCO.1998.16.6.2221

[B156] Cao Q Lu X Feng YJ Glycogen synthase kinase-3beta positively regulates the proliferation of human ovarian cancer cells Cell Res 2006 16 671 677 10.1038/sj.cr.7310078 16788573

[B157] Sun A Shanmugam I Song J Terranova PF Thrasher JB Li B Lithium suppresses cell proliferation by interrupting E2F-DNA interaction and subsequently reducing S-phase gene expression in prostate cancer Prostate 2007 67 976 988 10.1002/pros.20586 17440966

[B158] Ghosh JC Altieri DC Activation of p53-dependent apoptosis by acute ablation of glycogen synthase kinase-3beta in colorectal cancer cells Clin Cancer Res 2005 11 4580 4588 10.1158/1078-0432.CCR-04-2624 15958644

[B159] Shakoori A Mai W Miyashita K Yasumoto K Takahashi Y Ooi A Kawakami K Minamoto T Inhibition of GSK-3 beta activity attenuates proliferation of human colon cancer cells in rodents Cancer Sci 2007 98 1388 1393 10.1111/j.1349-7006.2007.00545.x 17640304 PMC11159717

[B160] Jordan CT The leukemic stem cell Best Pract Res Clin Haematol 2007 20 13 18 10.1016/j.beha.2006.10.005 17336250 PMC1988840

[B161] Ougolkov AV Fernandez-Zapico ME Bilim VN Smyrk TC Chari ST Billadeau DD Aberrant nuclear accumulation of glycogen synthase kinase-3beta in human pancreatic cancer: association with kinase activity and tumor dedifferentiation Clin Cancer Res 2006 12 5074 5081 10.1158/1078-0432.CCR-06-0196 16951223 PMC2692690

[B162] Garcea G Manson MM Neal CP Pattenden CJ Sutton CD Dennison AR Berry DP Glycogen synthase kinase-3 beta; a new target in pancreatic cancer? Curr Cancer Drug Targets 2007 7 209 215 10.2174/156800907780618266 17504118

[B163] Mamaghani S Patel S Hedley DW Glycogen synthase kinase-3 inhibition disrupts nuclear factor-kappaB activity in pancreatic cancer, but fails to sensitize to gemcitabine chemotherapy BMC Cancer 2009 9 132 10.1186/1471-2407-9-132 19405981 PMC2685435

[B164] Zhou H Tang Y Liang X Yang X Yang J Zhu G Zheng M Zhang C RNAi targeting urokinase-type plasminogen activator receptor inhibits metastasis and progression of oral squamous cell carcinoma in vivo Int J Cancer 2009 125 453 462 10.1002/ijc.24360 19391133

[B165] Lage H Therapeutic potential of RNA interference in drug-resistant cancers Future Oncol 2009 5 169 185 10.2217/14796694.5.2.169 19284376

[B166] Mai W Kawakami K Shakoori A Kyo S Miyashita K Yokoi K Jin M Shimasaki T Motoo Y Minamoto T Deregulated GSK3{beta} sustains gastrointestinal cancer cells survival by modulating human telomerase reverse transcriptase and telomerase Clin Cancer Res 2009 15 6810 6819 10.1158/1078-0432.CCR-09-0973 19903789

[B167] Weinstein IB Cancer. Addiction to oncogenes--the Achilles heal of cancer Science 2002 297 63 64 10.1126/science.1073096 12098689

[B168] Ding Q He X Xia W Hsu JM Chen CT Li LY Lee DF Yang JY Xie X Liu JC Hung MC Myeloid cell leukemia-1 inversely correlates with glycogen synthase kinase-3beta activity and associates with poor prognosis in human breast cancer Cancer Res 2007 67 4564 4571 10.1158/0008-5472.CAN-06-1788 17495324

[B169] Farago M Dominguez I Landesman-Bollag E Xu X Rosner A Cardiff RD Seldin DC Kinase-inactive glycogen synthase kinase 3beta promotes Wnt signaling and mammary tumorigenesis Cancer Res 2005 65 5792 5801 10.1158/0008-5472.CAN-05-1021 15994955

[B170] Dong J Peng J Zhang H Mondesire WH Jian W Mills GB Hung MC Meric-Bernstam F Role of glycogen synthase kinase 3beta in rapamycin-mediated cell cycle regulation and chemosensitivity Cancer Res 2005 65 1961 1972 10.1158/0008-5472.CAN-04-2501 15753396

[B171] Dal Col J Dolcetti R GSK-3beta inhibition: at the crossroad between Akt and mTOR constitutive activation to enhance cyclin D1 protein stability in mantle cell lymphoma Cell Cycle 2008 7 2813 2816 18769147 10.4161/cc.7.18.6733

[B172] Wang Y Lam JB Lam KS Liu J Lam MC Hoo RL Wu D Cooper GJ Xu A Adiponectin modulates the glycogen synthase kinase-3beta/beta-catenin signaling pathway and attenuates mammary tumorigenesis of MDA-MB-231 cells in nude mice Cancer Res 2006 66 11462 11470 10.1158/0008-5472.CAN-06-1969 17145894

[B173] Morrison JA Gulley ML Pathmanathan R Raab-Traub N Differential signaling pathways are activated in the Epstein-Barr virus-associated malignancies nasopharyngeal carcinoma and Hodgkin lymphoma Cancer Res 2004 64 5251 5260 10.1158/0008-5472.CAN-04-0538 15289331

[B174] Zheng H Saito H Masuda S Yang X Takano Y Phosphorylated GSK3beta-ser9 and EGFR are good prognostic factors for lung carcinomas Anticancer Res 2007 27 3561 3569 17972518

[B175] Mai W Miyashita K Shakoori A Zhang B Yu ZW Takahashi Y Motoo Y Kawakami K Minamoto T Detection of active fraction of glycogen synthase kinase 3beta in cancer cells by nonradioisotopic in vitro kinase assay Oncology 2006 71 297 305 10.1159/000106429 17652946

[B176] Ougolkov AV Fernandez-Zapico ME Savoy DN Urrutia RA Billadeau DD Glycogen synthase kinase-3beta participates in nuclear factor kappaB-mediated gene transcription and cell survival in pancreatic cancer cells Cancer Res 2005 65 2076 2081 10.1158/0008-5472.CAN-04-3642 15781615

[B177] Ougolkov AV Billadeau DD Targeting GSK-3: a promising approach for cancer therapy? Future Oncol 2006 2 91 100 10.2217/14796694.2.1.91 16556076

[B178] Bilim V Ougolkov A Yuuki K Naito S Kawazoe H Muto A Oya M Billadeau D Motoyama T Tomita Y Glycogen synthase kinase-3: a new therapeutic target in renal cell carcinoma Br J Cancer 2009 101 2005 2014 10.1038/sj.bjc.6605437 19920820 PMC2795437

[B179] Abrahamsson AE Geron I Gotlib J Dao KH Barroga CF Newton IG Giles FJ Durocher J Creusot RS Karimi M Glycogen synthase kinase 3beta missplicing contributes to leukemia stem cell generation Proc Natl Acad Sci USA 2009 106 3925 3929 10.1073/pnas.0900189106 19237556 PMC2646624

[B180] Ougolkov AV Bone ND Fernandez-Zapico ME Kay NE Billadeau DD Inhibition of glycogen synthase kinase-3 activity leads to epigenetic silencing of nuclear factor kappaB target genes and induction of apoptosis in chronic lymphocytic leukemia B cells Blood 2007 110 735 742 10.1182/blood-2006-12-060947 17463171 PMC1924475

[B181] Wang Z Smith KS Murphy M Piloto O Somervaille TC Cleary ML Glycogen synthase kinase 3 in MLL leukaemia maintenance and targeted therapy Nature 2008 455 1205 1209 10.1038/nature07284 18806775 PMC4084721

[B182] Adler JT Cook M Luo Y Pitt SC Ju J Li W Shen B Kunnimalaiyaan M Chen H Tautomycetin and tautomycin suppress the growth of medullary thyroid cancer cells via inhibition of glycogen synthase kinase-3beta Mol Cancer Ther 2009 8 914 920 10.1158/1535-7163.MCT-08-0712 19372564 PMC2670470

[B183] Kotliarova S Pastorino S Kovell LC Kotliarov Y Song H Zhang W Bailey R Maric D Zenklusen JC Lee J Fine HA Glycogen synthase kinase-3 inhibition induces glioma cell death through c-MYC, nuclear factor-kappaB, and glucose regulation Cancer Res 2008 68 6643 6651 10.1158/0008-5472.CAN-08-0850 18701488 PMC2585745

